# Genotoxicity Studies on Carrageenan: Short-term *In Vitro* Assays

**DOI:** 10.5487/TR.2009.25.1.051

**Published:** 2009-03-01

**Authors:** Young-Shin Chung, Ki-Hwan Eum, Seon-A Choi, Se-Wook Oh, Sue Nie Park, Young-Na Yum, Joo-Hwan Kim, Young-Rok Seo, Michael Lee

**Affiliations:** 19 Medvill Co., Ltd., Gasan-dong, Geumcheon-gu, Seoul, Korea; 29 Department of Biology, College of Natural Sciences, University of Incheon, 177 Dowha-dong, Nam-gu, Incheon, 402-749 Korea; 39 Korea Food Research Institute, Sungnam, Korea; 49 Division of Genetic Toxicology, National Institute of Toxicological Research, Korea Food and Drug Administration, Korea; 59 Department of Pharmacology, Institute for Basic Medical Science, Kyung Hee University, Seoul, Korea

**Keywords:** Carrageenan, Ames assay, Chromosomal aberration test, Micronucleus assay, Comet assay, Genotoxicity

## Abstract

Carrageenan is a naturally-occurring sulfated polygalactan which has been widely used in the dairy industry and a gelling agent in non-dairy products. In this study, four short-term *in vitro* genotoxicity assays were investigated to evaluate the potential genotoxic effects of carrageenan. The mutagenic-ity of carrageenan was evaluated up to a maximum dose of 5 mg/plate in Ames test. There was no increase in the number of revertant colonies compared to its negative control at any dose in all of strains tested. To assess clastogenic effect, the *in vitro* chromosomal aberration assay was performed using Chinese hamster lung cells. Carrageenan was not considered to be clastogenic in this assay at up to the highest feasible concentration which could be evaluated. The *in vitro* comet assay and micronucleus test results obtained on L5178Y cells also revealed that carrageenan has no genotoxicity potential, although there was a marginal increase in micronuclei frequencies and DNA damage in the respective micronucleus and comet assays. Taken together, our results indicate that carrageenan was not genotoxic based on four *in vitro* genotoxicity results.

## INTRODUCTION

Food-grade carrageenans are carbohydrate polymers derived from several species of red seaweed of the class *Rhodophyceae* and is being used particularly in dairy products as a stabilizer, gelling agent, and viscosity control agent. Carrageenan has been known to be not absorbed from the gastrointestinal tract, and be not degraded or fermented in the gut (JECFA, 1999). The long safe history of carrageenan was confirmed by negative results in subchronic and chronic feeding studies in many animal species (IARC, 1983; Weiner, 1991). It is well also documented that carrageenan does not show any genotoxicity in various *in vitro* and *in vivo* assays (JECFA, 1999; Weiner, 1991). Especially, carrageenan was found to be negative in the *Salmonella* mutagenicity test and unscheduled DNA synthesis (Mori *et al*., 1984; Prival *et al*., 1991).

However, the available data on the carcinogenicity of carrageenan are still controversial. Although many reports have demonstrated that carcinogenic activity of carrageenan has been evaluated as negative in mice, rats, and hamsters (Hagiwara *et al*., 2001; IARC, 1983; JECFA, 1999; Rustia *et al*., 1980), conflicting evidence also suggested that carrageenan might exert tumor promoting potential on colon carcinogenesis. Watanabe *et al*. (Watanabe *et al*., 1978) firstly reported that the dietary undegraded carrageenan had an enhancing effect in colorectal carcinogenesis in rats evoked by azoxymethane or methylnitrosourea. The several laboratories also confirmed the tumor promoting potential of carrageenan in the growth of aberrant crypt foci in the rat colon (Arakawa *et al*., 1988; Corpet *et al*., 1997). Conversely, the reports by Hagiwara *et al*. (2001) demonstrated that carrageenan lacks tumor promoting potential on 1,2-dimethylhydrazine-induced colorectal carcinogenesis in male F344 rats.

Thus, to investigate the whole genotoxic profile of carrageenan used as a food additive in Korea, we carried out the bacterial reverse mutation test (Ames test), *in vitro* chromosome aberration test using CHL cells, *in vitro* micronucleus test and comet assay using L5178Y cells.

## MATERIALS AND METHODS

***Test article and chemicals.*** Carrageenan in circulation in Korea was purchased at a local marketplace. Chemical and microorganism inspection on element standard of carrageenan was performed by Korea Advanced Food Research Institute (Seoul, Korea) according to Korean Food Additives Code. Carrageenan was dissolved in distilled water and serially diluted to the appropriate concentrations immediately before use. Most chemicals including positive controls such as 4-nitroquinoline 1-oxide (NQO) and cyclophosphamide were obtained from Sigma (St. Louis, MO USA). MEM medium, RPMI1640 medium, fetal bovine serum, and penicillin-streptomycin were purchased from GIBCO-Invitrogen (Carlsbad, CA). S9, which was prepared from male Sprague-Dawley rats induced with Aroclor 1254, was from Molecular Toxicology Inc. (Boone, NC) and cofactor for S9 mix was from Wako Pure Chem. Ind., Ltd. (Japan).

***Bacterial reverse mutation assay.*** *Salmonella typhimurium* strains TA98 and TA1537 (detect frameshift mutagens), and strains TA100, TA1535 and *Escherichia coli* WP2 *uvrA* (detect base-pair substitution mutagens) were used as tester strains. All of the test strains were purchased from Molecular Toxicology Inc. (Boone, NC). The mutation assay was performed according to the method of Chung *et al.* (2004), and Maron and Ames (1983). A 0.1 ml aliquot of carrageenan containing 156.3~5,000 μg per plate, 0.5 ml of S9 mix (or sodium-phosphate buffer, pH 7.4 for S9 negative group), and 0.1 ml inoculum of the tester strain were added to each tube containing 2 ml of top agar. The mixtures were poured onto the Vogel-Bonner minimal agar plates. Plates were incubated at 37°C for 48 h. Triplicate plates were run for each assay.

***In vitro chromosomal aberration assay.*** *In vitro* chromosomal aberration assay was performed using Chinese hamster lung fibroblast cells (CHL) (ATCC #CRL-1935), which were obtained from American Type Culture Collection (ATCC, Manassas, VA), as described by Hong *et al.* (2005), and Dean and Danford (1984) with minor modifications. The assay was consisted of short-term (6 h) and continuous (24 h) treatments. Approximately 22 hours after the start of the treatment, colcemid was added to each culture at a final concentration of 0.25 μg/ml. The slides of CHL cells were prepared following the hypotonic-methanol-glacial acetic acid-flame drying-Giemsa schedule for metaphase plate analysis. The 200 metaphases (100 metaphases from each duplicate culture) were selected and analyzed for each treatment group under 1000 × magnification using a light microscope. The results were expressed as mean aberrant metaphases excluding gaps per 100 metaphases. A valid test required the aberration frequencies of the solvent controls to be within the historical range of the laboratory.

***In vitro micronucleus test.*** The micronucleus assay was performed according to Kirsch-Volders *et al.* (2003) with modifications (Oliver *et al.*, 2006). The L5178Y $tk^{+/-}$. cell line (ATCC #CRL-9518, subclone 3.7.2-C) used in this study was provided by ATCC. The day before treatment, L5178Y mouse lymphoma cells were seeded at $2 \times 10^5$ cells/ml. Cells were treated with carrageenan for 3 h and harvested after a 21-h recovery period, or treated for 24-h and harvested immediately. The cellular suspension was centrifuged at 1000 rpm for 5 min and cells were then resuspended in a KCl 0.075 M solution maintained at room temperature for 10 min (mild hypotonic treatment). The fixation step with methanol/acetic acid (3 : 1) solution was repeated twice and finally, cells were resuspended in a small volume of methanol/acetic acid and dropped on to clean slides. The slides were stained with 10% Giemsa (pH 6.8). Relative growth was used to assess cytotoxicity, and no cells with reduced cell growth > 20% were scored. Micronuclei were counted in 2000 cells with well-preserved cytoplasm. For a valid test, the negative control had to have < 5%. Mitomycin C and colchicine were used as direct-acting positive controls, and cyclophosphamide was used in the presence of S9 as an indirect-acting positive control. The identification of micronuclei was carried out according to Fenech (2000).

***In vitro comet assay.*** Exponentially growing L5178Y $tk^{+/-}$. cells were seeded at $2 \times 10^5$ cells in 12-well plates and cultured for 24 h prior to carrageenan treatment, which was carried out for either 3 h or 24 h with the indicated concentrations of 50, 100 and 200 μg/ml. Following carrageenan treatment, cells were rinsed twice and resuspended at $2 \times 10^5$ cells/ml in ice-cold PBS. The comet assay was performed as described by Kim *et al.* (2006) and manufacturer's instruction. Briefly, cell suspension (25 μl) was mixed 1 : 10 with 250 μl molten low melting point (LMP) agarose, and samples of 75 μl of the mixture were rapidly spread on CometSlide™

(Trevigen, Gaithersburg, MD). After gelling for 20 min at 4°C in the dark, slides were put in a tank filled with lysis solution (2.5 M NaCl, 0.1 M EDTA, 10 mM Tris base, 1% sodium lauryl sarcosinate and 1% Triton X-100) for 1 h at 4°C in the dark. Slides were then washed three times with neutralization buffer (0.4 M Tris, pH 7.5) for 5 min and incubated in fresh alkaline buffer (0.3 M NaOH and 1 mM EDTA, pH > 13) for 30 min at room temperature to allow unwinding of DNA. Electrophoresis was then carried out at room temperature in fresh ice-cold alkaline electrophoresis buffer for 30 min (1 V/cm; 300 mA). After electrophoresis, slides were gently washed three times for 5 min in fresh neutralization buffer and exposed to 70% ethanol for 5 min. After drying at room temperature, slides were stained with 25 μl of ethidium bromide solution (20 μg/ml). Comets were examined at 200 × magnification using a fluorescence microscope (excitation filter, 515 560 nm; barrier filter, 590 nm) connected to a CCD camera. Images of 25 randomly selected nuclei per slide (two slides/culture, duplicate/dose) were analyzed using image-analysis software (Komet 5.0, Kinetic Imaging, Liverpool, UK). Tail Intensity (% of tail DNA) was used as the measure of DNA damage. The results are expressed as the mean ± SD (standard deviation). Methyl methanesulfonate was the direct-acting positive control, and cyclophosphamide was the indirect-acting positive control.

***Statistical analysis.*** The statistical analyses for *in vitro* chromosomal aberration and *in vitro* micronucleus results were conducted using Statistical Analysis System (SAS) program according to Richardson *et al.* (1989). A significant increase in micronuclei at any one concentration was determined based on a $P < 0.05$ from a one-tailed Fisher's exact test pair-wise comparison of each treatment group to control. A concentration-related response was determined based on a $P < 0.05$ from a one-tailed trend test. In CA test, pair-wise analyses of the percent aberrant cells in treated and control cultures were performed using Fisher's exact test. The result was judged as positive when there was a statistically significant and dose-related increase or a reproducible increase in the frequency of micronucleated cells (*in vitro* MN assay) or aberrant metaphases (*in vitro* CA assay). Statistically significant values that did not exceed the range of historic solvent control values were not considered positive. For statistical analysis of comet assay, the homogeneity of variances of data was tested with Bartlett's test ($P < 0.05$). If the variances of data were not equal, nonparametric Kruskal-Wallis test was used for statistical evaluations ($P < 0.05$ and 0.01). In case of Ames test, the previous results (McCann *et al.*, 1984) reported that statistical tests tend to identify more experiments as positive than considering the linear dose-response and a 2-fold increase over the spontaneous background for significance. The OECD Test Guideline 471 (1997) also suggested that biological relevance of the results should be considered first and statistical methods might be used as an aid in evaluating the test results. Thus, no statistical analysis was performed on Ames results.

## RESULTS

***Ames assay of carrageenan.*** Since no toxicity was observed after the addition of carrageenan up to 5,000 μg/plate in any strains, the mutagenicity of carrageenan was evaluated up to a maximum dose of 5,000 μg/plate at which the precipitation of test article was observed. The results of Ames test of carrageenan were shown in Table 1. There was no increase in number of revertant colonies compared to its vehicle control at any dose in all of strains. In addition, the antibacterial effects such as decrease in the number of colonies were not observed in all of strains.

***In vitro chromosomal aberration assay of carrageenan.*** *In vitro* CA test was performed with a high concentration level of 600 μg/ml due to precipitation of carrageenan in the vehicle at higher concentrations. The results of the *in vitro* chromosomal aberration assay of carrageenan are shown in Table 2. In the case of continuous treatment, total aberrations excluding gaps of carrageenan were less than 0.5% in CHL cells up to 600 μg/ml. In the short-term treatment, total aberrations excluding gaps of carrageenan were less than 0.5% without S-9 mix and less than 1% with S-9 mix, respectively. Since structural aberrations did not exceed 2% in either treatment, carrageenan was concluded to be non-clastogenic in this assay at up to the highest feasible concentration which could be evaluated. As expected, there was a significant increase in the number of aberrant metaphase both in the positive controls, cyclophosphamide (CPA) treatment for 6h+S and mitomycin C (MMC) treatment for 6h-S and 24h-S.

***In vitro micronucleus assay of carrageenan.*** Table 3 shows the results related to the evaluation of carrageenan in *in vitro* micronucleus test, which was performed with a high concentration level of 200 μg/ml due to the viscosity of carrageenan at higher concentrations. No cytotoxicity was observed whatever the concentration and the treatment schedule up to 200 μg/ml of carrageenan. Although there was a marginal increase

**Table 1.** Ames assay

| Tester strain | Chemical treated | Dose (μg/plate) | Without S-9 mix | With S-9 mix |
|---|---|---|---|---|
| | | | Colonies/plate (Mean) [Factor][a)] | Colonies/plate (Mean) [Factor] |
| TA100 | WSF | 0 | 118 ± 12 [1.0] | 93 ± 2 [1.0] |
| | | 156.3 | 119 ± 4 [1.0] | 135 ± 19 [1.5] |
| | | 312.5 | 104 ± 9 [0.9] | 119 ± 9 [1.3] |
| | | 625 | 110 ± 3 [0.9] | 125 ± 20 [1.3] |
| | | 1250 | 137 ± 13 [1.2] | 113 ± 18 [1.2] |
| | | 2500 | 113 ± 4 [1.0] | 103 ± 9 [1.1] |
| | | 5000# | 107 ± 4 [0.9] | 113 ± 17 [1.2] |
| TA1535 | WSF | 0 | 10 ± 2 [1.0] | 10 ± 3 [1.0] |
| | | 156.3 | 13 ± 5 [1.3] | 10 ± 3 [1.0] |
| | | 312.5 | 13 ± 0 [1.3] | 11 ± 2 [1.1] |
| | | 625 | 14 ± 3 [1.4] | 10 ± 5 [1.0] |
| | | 1250 | 17 ± 0 [1.7] | 9 ± 1 [0.9] |
| | | 2500 | 9 ± 4 [0.9] | 11 ± 5 [1.1] |
| | | 5000# | 8 ± 1 [0.8] | 14 ± 6 [1.4] |
| TA98 | WSF | 0 | 21 ± 7 [1.0] | 43 ± 5 [1.0] |
| | | 156.3 | 20 ± 6 [1.0] | 36 ± 3 [0.8] |
| | | 312.5 | 17 ± 1 [0.8] | 36 ± 7 [0.8] |
| | | 625 | 16 ± 6 [0.8] | 43 ± 2 [1.0] |
| | | 1250 | 24 ± 13 [1.1] | 38 ± 7 [0.9] |
| | | 2500 | 18 ± 1 [0.9] | 35 ± 12 [0.8] |
| | | 5000# | 21 ± 2 [1.0] | 43 ± 6 [1.0] |
| TA1537 | WSF | 0 | 6 ± 0 [1.0] | 5 ± 3 [1.0] |
| | | 156.3 | 1 ± 1 [0.2] | 7 ± 1 [1.4] |
| | | 312.5 | 5 ± 1 [0.8] | 6 ± 0 [1.2] |
| | | 625 | 3 ± 2 [0.5] | 8 ± 2 [1.6] |
| | | 1250 | 5 ± 1 [0.8] | 8 ± 6 [1.6] |
| | | 2500 | 9 ± 2 [1.5] | 6 ± 3 [1.2] |
| | | 5000# | 3 ± 2 [0.5] | 5 ± 2 [1.0] |
| *E. coli* | WSF | 0 | 42 ± 3 [1.0] | 42 ± 9 [1.0 |
| WP2 uvrA | | 156.3 | 46 ± 7 [1.1] | 53 ± 6 [1.3] |
| | | 312.5 | 37 ± 3 [0.9] | 51 ± 3 [1.2] |
| | | 625 | 45 ± 5 [1.1] | 53 ± 5 [1.3] |
| | | 1250 | 48 ± 2 [1.1] | 50 ± 7 [1.2] |
| | | 2500 | 45 ± 2 [1.1] | 48 ± 6 [1.1] |
| | | 5000# | 41 ± 10 [1.0] | 49 ± 13 [1.2] |
| TA100 | SA | 1.0 | 336 ± 14 [2.8] | |
| TA1535 | SA | 1.0 | 302 ± 30 [30.2] | |
| TA98 | 2-NF | 1.0 | 162 ± 9 [7.7] | |
| TA1537 | 9-AA | 80 | 747 ± 111 [124.5] | |
| WP2 uvrA | AF-2 | 0.01 | 132 ± 21 [3.1] | |
| TA100 | 2-AA | 1.0 | | 539 ± 55 [5.8] |
| TA1535 | 2-AA | 2.0 | | 156 ± 5 [15.6] |
| TA98 | 2-AA | 0.5 | | 190 ± 33 [4.4] |
| TA1537 | 2-AA | 2.0 | | 144 ± 21 [28.8] |
| WP2 uvrA | 2-AA | 20.0 | | 388 ± 14 [9.2] |

[a)]No. of colonies of treated plate/No. of colonies of negative control plate.
SA, Sodium azide; 9-AA, 9-Aminoacridine; 2-AA, 2-Aminoanthracene; 2-NF, 2-Nitrofluorene; AF-2, 2-Aminofluorene.
#Visible precipitation observed when added to top agar and at the end of treatment.

in the number of micronucleated cells, the difference was not statistically significant. Thus, carrageenan was classified as negative in all treatment schedules up to 200 μg/ml. The positive controls (CPA, MMC and colchicine) included in the study could induce a statistically significant increase in the number of micronucleated cells when compared to negative control group.

***In vitro* comet assay.** Comet assay was performed using a standard 3 h exposure time in both the absence

**Table 2.** *In vitro* chromosomal aberration assay

| Conc. (μg/ml) | S9 mix | Times[a)] (hours) | Aberrant metaphases excluding gaps (%) | Aberrant metaphases including gaps (%) |
|---|---|---|---|---|
| 6 h treatment | | | | |
| 0 | + | 6-18 | 0.5[b)] | 1.5 |
| 150 | + | 6-18 | 1.0 | 1.5 |
| 300 | + | 6-18 | 0.5 | 0.5 |
| 600[#] | + | 6-18 | 0.0 | 2.5 |
| CPA 5 | + | 6-18 | 36.5**[c)] | 38.0 |
| 6 h treatment | | | | |
| 0 | - | 6-18 | 0.0 | 1.5 |
| 150 | - | 6-18 | 0.5 | 1.0 |
| 300 | - | 6-18 | 0.0 | 0.5 |
| 600[#] | - | 6-18 | 0.0 | 2.0 |
| MMC 0.1 | - | 6-18 | 10.5** | 12.5 |
| 24 h treatment | | | | |
| 0 | - | 24-0 | 0.0 | 0.5 |
| 150 | - | 24-0 | 0.5 | 1.5 |
| 300 | - | 24-0 | 0.5 | 2.5 |
| 600[#] | - | 24-0 | 0.5 | 1.0 |
| MMC 0.1 | - | 24-0 | 28.0** | 33.5 |

[a)]Time, Chemical treatment time-recovery time.
[b)]Means of duplicate cultures; 100 metaphases were examined per culture.
[c)]Fisher's exact test; ** significantly different from the control at $P < 0.01$.
[#]Visible precipitation observed when treated and at the end of the treatment.
Abbreviation: CPA, cyclophosphamide monohydrate; MMC, mitomycin C.

**Table 3.** *In vitro* micronucleus assay

| Conc. (μg/ml) | S9 mix | Times[a)] (hours) | Mean micronuclei/ 1000 cells[b)] | Relative cell count (%) |
|---|---|---|---|---|
| 3 h treatment | | | | |
| 0 | + | 3-21 | 18.5 | 100 |
| 50 | + | 3-21 | 26.5 | 80 |
| 100 | + | 3-21 | 25.5 | 83 |
| 200 | + | 3-21 | 30.0 | 85 |
| CPA (5 μg/ml) | + | 3-21 | 64.5**[d)] | 64 |
| 3 h treatment | | | | |
| 0 | - | 3-21 | 21.0 | 100 |
| 50 | - | 3-21 | 30.5 | 94 |
| 100 | - | 3-21 | 31.5 | 86 |
| 200 | - | 3-21 | 31.5 | 94 |
| MMC (0.125 μg/ml) | - | 3-21 | 148.0** | 80 |
| COL (0.2 μg/ml) | - | 3-21 | 58.5** | 54 |
| 24 h treatment | | | | |
| 0 | - | 24-0 | 18.5 | 100 |
| 50 | - | 24-0 | 24.0 | 109 |
| 100 | - | 24-0 | 31.0 | 112 |
| 200 | - | 24-0 | 21.5 | 113 |
| MMC (0.0625 μg/ml) | - | 24-0 | 155.0** | 89 |
| COL (0.2 μg/ml) | - | 24-0 | 117.0** | 40 |

[a)]Time, Chemical treatment time-recovery time.
[b)]2000 cells were examined per culture.
[c)]RCC = (Cell counts of treated flask/Cell counts of untreated flask) × 100.
[d)]Fisher's exact test; $**P < 0.01$.
Abbreviation: RCC, Relative cell count; CPA, cyclophosphamide; MMC, mitomycin C; COL, colchicine.

**Table 4.** *In vitro* comet assay

| Conc. (μg/ml) | S9 mix | % Tail DNA[a)] | Olive tail moment | Relative cell count (%)[b)] |
|---|---|---|---|---|
| 3 h treatment | | | | |
| 0 | + | 7.86 ± 3.91 | 5.51 ± 3.23 | 100 |
| 50 | + | 8.44 ± 4.48 | 7.47 ± 6.84 | 84.2 |
| 100 | + | 8.98 ± 5.11 | 6.53 ± 3.66 | 77.4 |
| 200 | + | 10.72 ± 4.99** | 7.31 ± 3.12 | 68.5 |
| 3 h treatment | | | | |
| 0 | - | 7.41 ± 4.55 | 5.14 ± 5.15 | 100 |
| 50 | - | 8.92 ± 4.99 | 6.63 ± 3.49 | 93.2 |
| 100 | - | 8.95 ± 4.61 | 6.43 ± 3.60 | 90.4 |
| 200 | - | 10.14 ± 5.34** | 7.63 ± 4.17 | 86.3 |
| 24 h treatment | | | | |
| 0 | - | 6.46 ± 3.85 | 4.41 ± 3.50 | 100 |
| 50 | - | 8.60 ± 4.87** | 6.36 ± 5.62 | 81.0 |
| 100 | - | 9.01 ± 4.64** | 6.47 ± 4.73 | 76.6 |
| 200 | - | 8.86 ± 5.25** | 7.36 ± 7.09 | 63.2 |
| Positive controls | | | | |
| CPA (10 μg/ml, 3 h) | + | 19.29 ± 12.54** | 11.51 ± 6.96 | 54.8 |
| $H_2O_2$ (200 μM, 30 min) | - | 88.59 ± 5.17** | 78.60 ± 15.37 | 71.1 |
| MMS (0.1 μM, 3 h) | - | 77.78 ± 6.15** | 48.54 ± 6.15 | 74.0 |

*$p < 0.05$, statistically significant vs. negative control according to Kruskal-Wallis test with post-test.
[a)]100 cells were examined per culture.
[b)]Viability measured by trypan blue exclusion; expressed as percentage of absorbance of control.

and presence of S9 mix for short treatment, and 24 h exposure time for continuous treatment with a high concentration level of 200 μg/ml due to the viscosity of carrageenan in the vehicle at higher concentrations. Cytotoxicity is evaluated in terms of decrease in cell viability immediately after treatment, using the Trypan blue exclusion test. With carrageenan, cell growth was not significantly affected in the range of concentrations tested. The results of the alkaline comet assay with L5178Y $tk^{+/-}$ cells were shown Table 4. Slight but statistically significant increases in % tail DNA were sporadically found. However, this marginal increase was not concentration-dependent and none exceeded a maximal fold increase of 2.0. Thus, carrageenan was judged to be negative in all the treatment schedules. The positive controls treated with CPA and MMS caused significant ($P < 0.01$) increases in % tail moment as well as Olive tail moment.

## DISCUSSION

The safety of carrageenan as a food additive is supported by many toxicological studies in animal species including rats, mice, guinea pigs, and monkeys at high levels in the diet (Weiner, 1991) since the commercial production of carrageenan in 1930s in America. Food-grade carrageenan was found to lack carcinogenicity in chronic feeding studies in rats and golden hamsters at levels up to 5% (Rustia *et al.*, 1980). Carrageenan also showed negative genotoxic responses in many genotoxicity tests including Ames test (Mori *et al.*, 1984; Prival *et al.*, 1991), dominant lethal assay, and cytogenetic assay in rats (Food and Drug Administration, 1972). However, there has arisen some questions that carrageenan might possess promoting effects on colon carcinogenesis. In co-carcinogenicity test, carrageenan enhanced the incidence of colon tumors in rats treated with known carcinogens (Arakawa *et al.*, 1988; Corpet *et al.*, 1997; Watanabe *et al.*, 1978). Regarding these positive results, Hagiwara *et al.* (2001) argued that their experiments were performed under unusual conditions, since animals were exposed to extremely high dietary levels which exceeded that accepted as the maximum dose (5%) for chronic studies. Thus, it is of interest to determine whether carrageenan used as a food additive in Korea contains detectable levels of cell mutagens. In this study, Ames test showed that there was no significant increase in the number of revertant colonies compared to its negative control at any dose in all strains.

Contrary to Ames test using bacterial strains, a little data are available in literature on the potential clastogenicity of carrageenan. Thus, potential clastogenic effects of carrageenan in three short-term *in vitro* genotoxicity assays using mammalian cells were investigated, which included chromosomal aberration assay, micronucleus test and the alkaline comet assay. Three *in vitro* genotoxicity assays were performed with the highest concentration of 200 μg/ml (MN test and comet assay), or 600 μg/ml (CA test) because of the viscosity of carrageenan at the higher concentrations. Carrageenan has been found to readily form viscous solutions at temperatures greater than the solubilization temperature due to the unbranched linear macromolecular structure (Weiner, 1991). There was little clastogenicity seen in the *in vitro* chromosomal aberration assay. Also, in the *in vitro* micronucleus test, carrageenan was classified as negative in all treatment schedules although there was a marginal increase in the number of micronucleated cells. Since the presence of cytochalasin B was shown to increase the levels of spontaneous micronucleated cells in the L5178Y cell line (Lorge *et al.*, 2006), *in vitro* MN test was performed without treatment of cytochalasin B, which is generally required to make sure analysed cells have completed a division (Fenech and Morley, 1985).

The alkaline (pH > 13) comet assay has been the most commonly practiced assay for the routine screening of potential genotoxicants (Rojas *et al.*, 1999; Tice *et al.*, 2000). While the micronucleus test detects DNA lesions after their fixation into chromosome mutations, the alkaline version of the comet assay (Singh *et al.*, 1988) detects primary DNA single and double strand breaks and alkali-labile sites. Table 4 shows the % tail DNA and Olive tail moment obtained in the alkaline comet assay with carrageenan (50, 100 and 200 μg/ml). There is much to recommend the use of % tail DNA, as this gives a clear indication of the appearance of the comets and is linearly related to the DNA break frequency over a wide range of levels of damage (Hartmann *et al.*, 2003; Burlinson *et al.*, 2007) although tail moment measurement is the most commonly reported. Thus, in this study, comet assay results were analyzed with % tail DNA. Nonparametric Kruskal-Wallis test was used for statistical evaluations of % tail DNA because there is no consensus on standard statistical methods for the analysis of comet data (Tice *et al.*, 2000). Carrageenan appeared to induce slight but statistically significant increases in % tail DNA. However, it is considered that there is no evidence of biological relevance because this marginal increase was not concentration-dependent and none exceeded a maximal fold increase of 2.0.

Taken together, the safety of carrageenan as a natural food additive was confirmed by negative results in four *in vitro* genotoxicity studies. In fact, strongly anionic structure of carrageenan is quite unlikely to interact with DNA. However, purity is a pre-requisite that can influence safety of food additives. For food applications, the Korean Food Additives Codex specifies the purity of commercial food-grade carrageenan (Korean Food Additives Codex, 2008). The ester sulfate content must be between 15.0% and 40.0% on a dry weight basis. Heavy metals, lead, arsenic, acid-insoluble ash, acid-insoluble matter, total ash and loss on drying are all specified. Thus, it will be important to manage the process of carrageenan production to ensure that there are no contaminants with genotoxic potentials.

## ACKNOWLEDGMENTS

This research was supported by a grant (08152NTP421) from Korea Food & Drug Administration in 2008 and the views presented in this article do not necessarily reflect those of the KFDA.
